# Using Rough Sets to Improve Activity Recognition Based on Sensor Data †

**DOI:** 10.3390/s20061779

**Published:** 2020-03-23

**Authors:** Hans W. Guesgen

**Affiliations:** School of Fundamental Sciences, Massey University, Palmerston North 4442, New Zealand; h.w.guesgen@massey.ac.nz; Tel.: +64-6-951-7137 (DDI)

**Keywords:** activity recognition, context awareness, spatial reasoning, rough sets

## Abstract

Activity recognition plays a central role in many sensor-based applications, such as smart homes for instance. Given a stream of sensor data, the goal is to determine the activities that triggered the sensor data. This article shows how spatial information can be used to improve the process of recognizing activities in smart homes. The sensors that are used in smart homes are in most cases installed in fixed locations, which means that when a particular sensor is triggered, we know approximately where the activity takes place. However, since different sensors may be involved in different occurrences of the same type of activity, the set of sensors associated with a particular activity is not precisely defined. In this article, we use rough sets rather than standard sets to denote the sensors involved in an activity to model, which enables us to deal with this imprecision. Using publicly available data sets, we will demonstrate that rough sets can adequately capture useful information to assist with the activity recognition process. We will also show that rough sets lend themselves to creating Explainable Artificial Intelligence (XAI).

## 1. Introduction

Most developed countries around the world are facing the problem of an aging population. Life expectation is higher than ever before, and so is the expectation to live a high-quality, independent lifestyle throughout the entire life, independent of age or illnesses such as Alzheimer’s or Parkinson’s disease. Unfortunately, this expectation is not always met. Rather than moving to a nursing home or employing the continuous support of a caretaker, we now have the technology to set up smart homes:

- To make sure the person living in the smart home is safe and is performing routine activities.
- To help the person in the smart home to compensate for impairments.
- To determine the physical or cognitive status of the person in the smart home.

Since activity recognition is an essential component of a smart home, research into activity recognition has gained significant attention over the last 20 years. Most approaches to activity recognition are based on using sensors, which monitor the person’s activities in a non-obtrusive way. The sensors produce a stream of data, which is then analyzed by an intelligent reasoning engine. The reasoning engine must determine which activity currently takes place and whether this activity is a normal behavior or poses a threat to the person living in the smart home.

Approaches to activity recognition range from logic-based approaches to probabilistic machine learning approaches [1,2,3,4,5,6,7,8,9,10,11,12]. Much progress has been made in recent years but determining the correct activity from sensor data alone is often impossible:

- Sensors can only provide very limited information.
- Human behavior is inherently complex.
- Activities may be interleaved.
- There is often more than one way to perform an activity.

As has been pointed out in [13], there is a wide range of sensors that can be used for activity recognition. We focus in this article on the least intrusive ones, which are stationary sensors providing binary data (e.g., motion sensors).

Several researchers have realized that context information can be useful to improve the activity recognition process [14,15,16,17]. Context information includes the time when an activity is performed, the location it is performed at, the circumstances under which it is performed, etc. For example, breakfast usually occurs in the kitchen in the morning, which means that if something is happening at 07:00 in the kitchen, it is more likely to be breakfast than taking a shower.

In this article, we focus on spatial context information and how it can be represented to improve the activity recognition process. Since we extract the spatial context information from sensor data, one might argue that this is not different from using this information in the recognition process in the first place [18]. However, the fundamental difference is that the spatial sensor data are preprocessed so that they more or less accurately represent the space in which the activity occurs. This also allows us to abstract from time (when is which space used during the activity), which compensates for any temporal variation in the activity.

Since the space used for a particular type of activity might slightly differ from one occurrence of the activity to another, we need to be able to represent vagueness in our approach. There are various ways to express vagueness, such as probabilities, fuzzy sets, values of belief, etc. Our research has shown that (classical) rough sets are particularly useful for this. They provide information in a human-readable form, rather than an encoding in a neural network for instance, and therefore they can easily be analyzed and verified. As such, they may be considered a form of Explainable Artificial Intelligence (XAI), which has gained significant attention in recent years.

## 2. Basic Notions

Activities can take place in different parts of the house but often there are preferred locations for particular activities. For example, cooking usually occurs in the kitchen, which means it always takes place roughly in the same (physical) space of the smart home. As a result of that, the space where an activity occurs can be an indication of what kind of activity it is. However, we do not know exactly the physical space in which the activity is occurring, but we can approximate this space.

There are various sensors that indirectly provide spatial information, such as motion sensors, switches that register when doors are opened, pressure mats installed in the floor, and so on. In most cases, the space they are associated with is not exactly defined. For example, a tall person might trigger a motion sensor at a slightly different location compared to a small person, or doors can be opened from different sides. Nevertheless, motion sensors give us some indication of the physical space involved.

In the following, we will use the term spatial marker to denote a sensor that provides some information about the space of the activity. The set of all spatial markers defines the universe we reason about:

The set of spatial markers $s_{1},\ldots ,s_{n}$ of a smart home is called the universe *U*. Two different markers $s_{i}$ and $s_{j}$, i≠j, may correspond to overlapping physical spaces. The physical space that a spatial marker corresponds to is not necessarily precisely defined.

Since activities usually trigger more than one spatial marker, we need to consider set of spatial markers, rather than individual ones. For example, when cooking in the kitchen, several kitchen motion sensors might get activated on top of various door sensors:

A subset *S* of *U* (i.e., an element of the powerset of *U*, $2^{U}$) is called a space. There is a one-to-one mapping between each occurrence of a particular activity and a space, but different occurrences of the same activity might be mapped to different spaces.

For our further discussions, it is necessary to make the following observations:

Two spaces $S_{1}$ and $S_{2}$ are called disjoint iff the intersection of $S_{1}$ and $S_{2}$ is empty (i.e., $S_{1}\cap S_{2}=\emptyset$). If two spaces are disjoint, then the physical spaces that they correspond to are not necessarily disjoint.

Two spaces $S_{1}$ and $S_{2}$ are called overlapping iff the intersection of $S_{1}$ and $S_{2}$ is not empty (i.e., $S_{1}\cap S_{2}\neq \emptyset$). If two spaces are overlapping, then the physical spaces that they correspond to are also overlapping.

**Figure 1:**
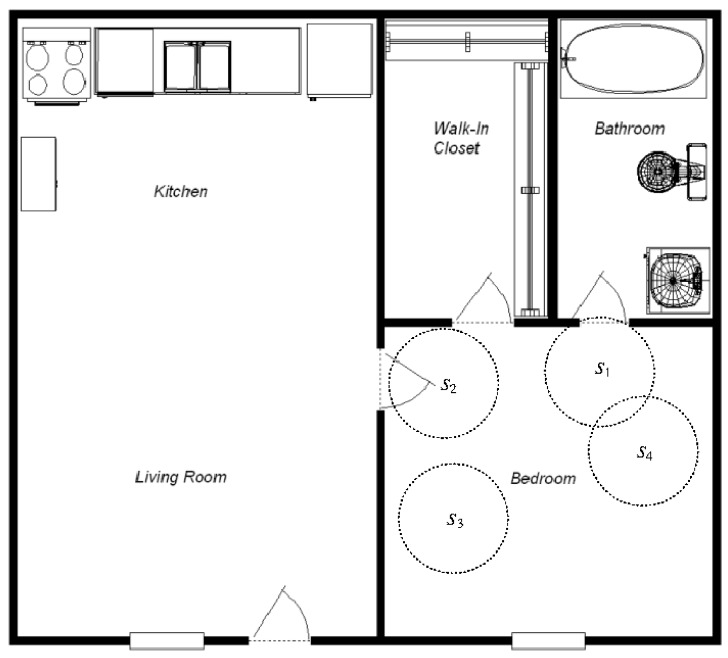
Floor plan of an apartment equipped with motion sensors.

Consider the floorplan of a fictional apartment in Figure 1, which has been equipped with motion sensors $s_{1}$, $s_{2}$, $s_{3}$, and $s_{4}$. The spaces $\left\{s_{1},s_{2}\right\}$ and $\left\{s_{2},s_{3}\right\}$ are overlapping, and so are their corresponding physical spaces, which are depicted by the dashed circles around the sensors. The spaces $\left\{s_{2}\right\}$ and $\left\{s_{3}\right\}$ are disjoint, and so are their corresponding physical spaces. However, the spaces $\left\{s_{1}\right\}$ and $\left\{s_{4}\right\}$ are disjoint, but the corresponding physical spaces are overlapping.

## 3. Activity Spaces

Since the space of each type of activity might differ from instance to instance, we need to abstract from particular instances of that type of activity. This involves a way of expressing vagueness when defining the space of the activity. For example, when cleaning the house, we might decide to thoroughly clean all rooms or we might decide to just clean the rooms that are getting dirty more easily. Depending on which rooms we choose, we get a larger or smaller space for the activity, and as a result of that, the type of activity needs to be represented by a concept that considers both the smaller and larger space.

A well-known theory for representing topological aspects of space in a qualitative way and for reasoning about them is the Region Connection Calculus [19]. Lehmann and Cohn [20] introduced an extension to RCC theory, called the egg-yolk theory, to express relationships between vague regions. Although we are not using the RCC theory in our approach, Lehmann and Cohn’s extension served as a blueprint for our approach. Their idea is to view a vague region as an egg consisting of two crisp regions: the yolk and the egg itself. All points within the yolk are considered to be in the region, whereas all points outside the egg are outside the region. The white (i.e., the egg without the yolk) characterizes the points that may or may not belong to the region.

There is a close relationship between the egg-yolk theory and rough sets [21]. In the following, we adopt notions from rough sets to characterize the space of a type of activity. Since the RCC theory, and consequently, the egg-yolk theory, is only concerned about topological relations, it does not represent the size of the regions. As a result, we are not able to make any judgement of how precisely defined a region is. This is different in our case, because spaces are defined in terms of spatial markers, which means they are finite sets. For vague sets based on spatial markers, we can define lower and upper approximations:

Let $S_{1},\ldots ,S_{m}$ be the spaces corresponding to some type of activity *A*, then $\underline{A}=S_{1}\cap \cdots \cap S_{m}$ (the intersection of all spaces used by *A*) is called the lower approximation of the activity space of *A*. Analogously, $\bar{A}=S_{1}\cup \cdots \cup S_{m}$ (the union of all spaces used by *A*) is called the upper approximation of the activity space of *A*.

The pair $\langle \underline{A},\bar{A}\rangle$ is called the activity space of *A*.

Considering again the floorplan shown in Figure 1, let us assume that we to describe the space used when a person sleeps in the bedroom. The sleeping activity starts when the person enters the bedroom and finishes when they leave the bedroom. Since it is possible to enter the bedroom through different doors, the spaces for the instances of sleeping may vary from each other. If the person enters the bedroom from the bathroom and leaves the bedroom the same way, the space associated with sleeping will be $\left\{s_{1},s_{3},s_{4}\right\}$, whereas if the person enters from the living room and leaves this way, the space will be $\left\{s_{2},s_{3},s_{4}\right\}$. If the person enters and leaves through different doors, the space will be $\left\{s_{1},s_{2},s_{3},s_{4}\right\}$. This means that the lower and upper approximations for this type of activity are $\left\{s_{3},s_{4}\right\}$ and $\left\{s_{1},s_{2},s_{3},s_{4}\right\}$, respectively.

The set-theoretic difference between the lower and upper approximation of an activity space (also referred to as the boundary space, to use the terminology from rough sets) provides us with some idea of how precisely defined the activity space is:

Let $\underline{A}$ and $\bar{A}$ be the lower and upper approximation of the space of a type of activity *A*, respectively. Then $\bar{A}/\underline{A}$ is called the boundary space of *A*.

If the boundary space of *A* is empty, then the activity space of *A* is precisely defined.

In the example, the boundary space is $\left\{s_{1},s_{2}\right\}$.

In general, the boundary space is not empty, unless there is no variation in the instances of a particular type of activity. The size of the boundary space provides some indication of the vagueness of the activity space. However, just taking the size can be misleading, as it is neither related to the size of the lower approximation nor to the size of the upper approximation of the activity space. For example, if there is only one motion sensor installed in each of the rooms of the house, an additional motion sensor activation is quite significant while this is not the case if rooms have many motion sensors. To overcome this problem, we adopt the notion of accuracy of rough sets:

Given a type of activity *A*, the accuracy of its activity space is defined as $\alpha \left(A\right)=\frac{|\underline{A}|}{|\bar{A}|}$.

The accuracy of an activity space can be interpreted as the ratio of the number of spatial markers that are necessarily involved in a type of activity to the number of spatial markers that may be involved in this type of activity. Its value is in the range between 0 (extremely vague) and 1 (precise). When the accuracy is high, the activity occurs in a more defined space than when the accuracy is low. The accuracy of the activity space in the example is $\frac{2}{4}=\frac{1}{2}$.

If the activity occurs in more or less the same physical space, computing the upper and lower approximations of an activity space is straightforward. For an activity like cooking, this is most likely the case, since it is usually confined to the kitchen. However, this might not hold for an activity like sleeping, because at night it probably occurs in the bedroom but during the day it might occur on the couch in the lounge. If the first scenario is the case, then we simply collect the spatial markers for each of the occurrences. The union of the markers then defines the upper approximation of the activity space while the intersection of the markers defines its lower approximation. Would we use the same approach in the second scenario where activities occur in completely different physical spaces, we would end up with approximations that might be too coarse. In this case, it makes sense to apply a hierarchical clustering algorithm to the spatial markers and compute the approximations for each of the clusters.

In a hierarchical clustering algorithm, each space is initially put into a cluster of its own, Then the two nearest clusters are repeatedly merged into one cluster until some termination criterion is reached. Spatial markers do not define a Euclidean space, which means that the centroid of a cluster is not well defined. Therefore, we cannot use the centroids to determine the distance between clusters. Instead, we apply the notion of cohesion, which means that we combine those two clusters whose union is most cohesive. Cohesion can be defined as the maximum distance between the spaces in the cluster. Most cohesive means that the maximum distance between the spaces is minimal.

To determine the distance between two spaces $S_{1}$ and $S_{2}$, we represent each space as a bit vector, which indicates whether a spatial marker is present in the space or not. We then use the Hamming distance to define the distance between the spaces. Given bit vectors for $S_{1}$ and $S_{2}$, the Hamming distance counts the positions in the two bit vectors where they differ from each other. Another way to compute the Hamming distance is to determine the cardinality of the set $\left(S_{1}\cup S_{2}\right)/\left(S_{1}\cap S_{2}\right)$.

## 4. Boosting Activity Recognition

Logic-based approaches to activity recognition require an extensive modelling process while machine learning approaches use an iterative process based on data obtained from a smart home. In either case, it is important not to increase the complexity of these approaches unnecessarily. In the ideal case, we want an algorithm with a constant time behavior, or in the case where the algorithm is based on a data set, an algorithm which is linear in the size of the data set. Since our approach is based on a data set, we aim to achieve the latter.

When the activity recognizer is trained, we record the spaces when data are presented to the machine learner. This is only for data points for which the ground truth is known, yielding activity spaces or sets of activity spaces for the most commonly occurring activities in the smart home. When the training is completed, and the machine learner is used for recognizing activities, we use the activity spaces to resolve ambiguities in the activity recognition process. For example, if two activities are equally plausible, looking at their activity spaces might lead to preferring one activity over the other. Or if there is not enough evidence to strongly support an activity, then the activity space of the activity might be used as additional support.

Let us assume that we have obtained activity spaces for a set of activities $\left\{A_{1},\ldots ,A_{n}\right\}$. For the sake of simplicity, we assume that each activity is associated with exactly one activity space, rather than a set of activity spaces. As before, we denote the activity space of $A_{i}$ as $\langle \underline{A_{i}},\bar{A_{i}}\rangle$ for i=1,…,n. Further let us assume that *B* is the current activity to be classified. We know the activity space of *B*, $\langle \underline{B},\bar{B}\rangle$, which is a crisp set (i.e., $\underline{B}=\bar{B}$). For convenience, we use $\underline{\bar{B}}$ to denote this set.

In the boosting process, we distinguish three cases:

1. $\underline{\bar{B}}$ is not a superset of any known lower approximation of an activity space ($\underline{A_{i}}⊈\underline{\bar{B}},i=1,\ldots ,n$). This means that we have not yet seen an activity that occupies the space used by *B*. Therefore, we cannot make any assumption about the type of activity that we have just observed.
2. $\underline{\bar{B}}$ is a superset of exactly one lower approximation ($\underline{A_{k}}\subseteq \underline{\bar{B}}$). This means that we have seen an activity before with a set of necessarily involved spatial markers that are also used by *B*. It is therefore, likely that *B* and $A_{k}$ are the same type of activity.
3. $\underline{\bar{B}}$ is a superset of *m* known lower approximations of activity spaces. ($\underline{A_{i_{j}}}\subseteq \underline{\bar{B}},i_{j}\in \left\{1,\ldots ,n\right\},j=1,\ldots ,m$). This means that we have several candidates for the type of activity of *B*. We can put them in an order of preference by comparing their upper approximations with the space used by *B*. The smaller the difference between the upper approximation of a known activity and $\underline{\bar{B}}$, the more likely it is that we have seen the activity before. This can be expressed in terms of the accuracy of the activity space $\langle \bar{A_{i_{j}}}\cap \underline{\bar{B}},\bar{A_{i_{j}}}\rangle$.

The guidelines above are heuristics and do not guarantee that they result in the correct activity. There are many factors that determine how accurate the predictions are. If the activity spaces are characteristic for each type of activity considered by the system, then there is a good chance that the correct type of activity is selected. This is more likely in dwellings with rooms dedicated to certain types of activities, like bedrooms for sleeping, kitchens for preparing meals and washing dishes, lounges for watching TV, and so on. In single room apartments this is most likely not the case, and as a result of that, activity spaces overlap significantly.

## 5. Evaluation

There are several data sets that can be used to evaluate approaches to activity recognition, even synthetic ones [22]. The CASAS data sets [23] are arguably the most popular ones, as they not only provide data from smart homes with single occupancy but also provide data from homes with multiple residents. In our study, we used the Aruba data set to evaluate our approach in the context of smart homes with one resident and the twor.2010 data set to study smart homes with two residents.

**Figure 2:**
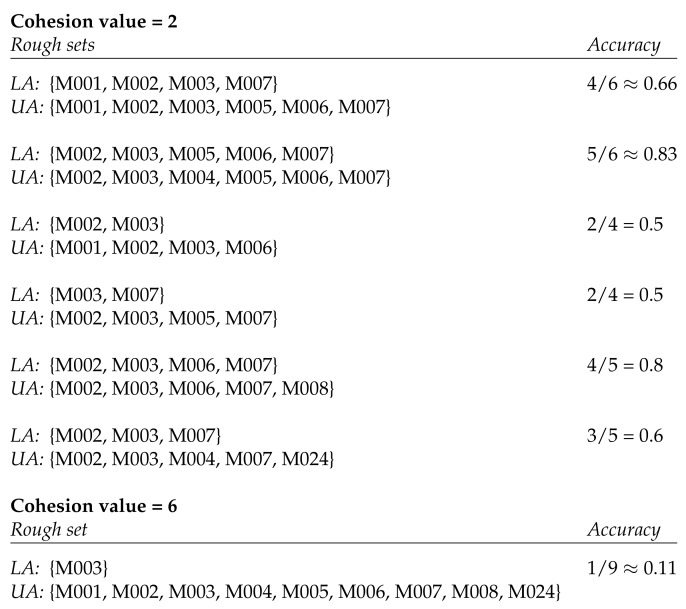
Rough sets resulting from clustering the activity spaces of sleeping with cohesion values 2 and 6. The spatial markers in the Aruba data set that correspond to motion sensors are denoted by ‘M’ and a three-digit number. *LA* denotes the lower approximation and *UA* the upper approximation of the rough set.

The Aruba data set contains 11 types of activities. We preprocessed the Aruba data set by computing the activity space for each type of activity recorded in the data set. We then clustered the activity spaces with cohesion values ranging from 2 to 20 and analyzed the resulting rough sets. Figure 2 shows an example obtained by clustering the activity spaces for sleeping with cohesion values 2 and 6. Permitting a maximal cohesion value of 2 results in 6 clusters whose rough sets have various degrees of accuracy. The most precise ones have an accuracy of 0.5, while the least precise ones have an accuracy of around 0.8. Allowing the cohesion value to go up to 6 would result in only one cluster. The rough set of that cluster has an accuracy of around 0.1.

A low cohesion values means that we get a high accuracy of the resulting rough sets. If we set the cohesion value to 0, we obtain an accuracy of 1. However, this means that each space is left in its own cluster and that the corresponding rough set of that cluster is the space itself. In other words, the rough set is in fact a crisp set in this case.

In homes with multiple residents, we are not only interested in what type of activity is taking place but also who is performing the activity [24]. This makes the problem of activity recognition more challenging and consequently boosting the activity recognition process more important. If the residents of the smart home perform a certain activity in exactly the same way, it is unlikely that we can determine who is performing the activity. However, if the residents perform the activity in different places, we might be able to distinguish between the residents.

**Figure 3:**
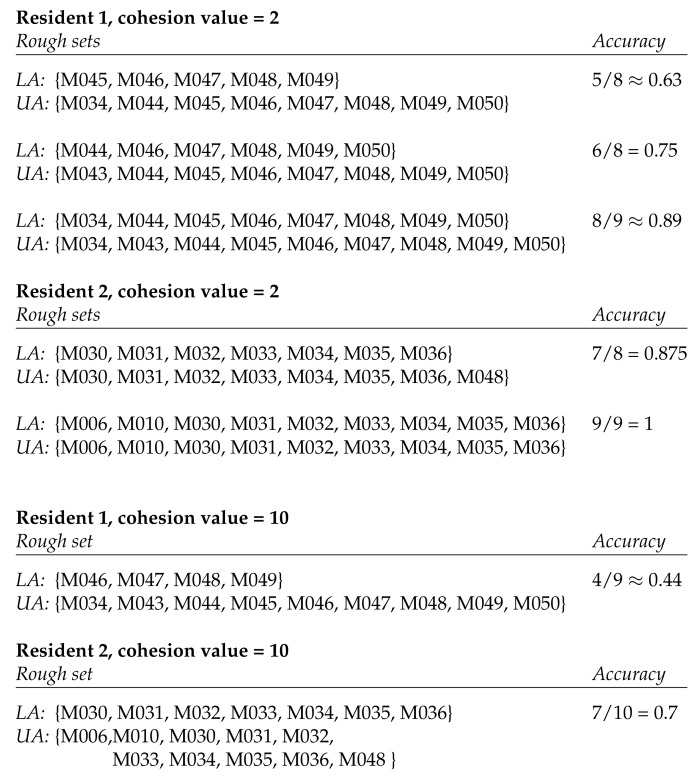
Rough sets resulting from clustering the activity spaces of two residents wandering in the smart home with cohesion values 2 and 10. The data set used for this analysis is the CASAS twor.2010 data set. *LA* denotes the lower approximation and *UA* the upper approximation of the rough set.

Figure 3 shows the rough sets that result from applying hierarchical clustering to the activity spaces involved in the twor.2010 data set from the CASAS suite. When using a cohesion value of 10, we obtain one rough set for each of the residents. They not only differ in the sets themselves but also in the accuracy of the sets. A cohesion value of 2 yields different numbers of rough sets for the residents. Again, these differ in both the sets themselves and their accuracies.

## 6. Implementation

We have implemented our approach in Python on a PC with an Intel(R) Core(TM) i7-7600U CPU @ 2.80GHz and 16GB of RAM. The data sets that we have used to evaluate our approach all have more or less the same format. They consist of a sequence of sensor tokens such as the following:

2010-11-04 02:32:33.351906 M003 ON

A sensor token consists of a date/time stamp, a sensor identifier, and a sensor state. Some sensor tokens are annotated with the name of an activity and a label that states whether the sensor token starts or finishes the activity:

2010-11-04 00:03:50.209589 M003 ON Sleeping begin

All sensor tokens between a begin and end bracket are considered to be caused by the activity named in the annotated sensor token.

**Figure 4:**
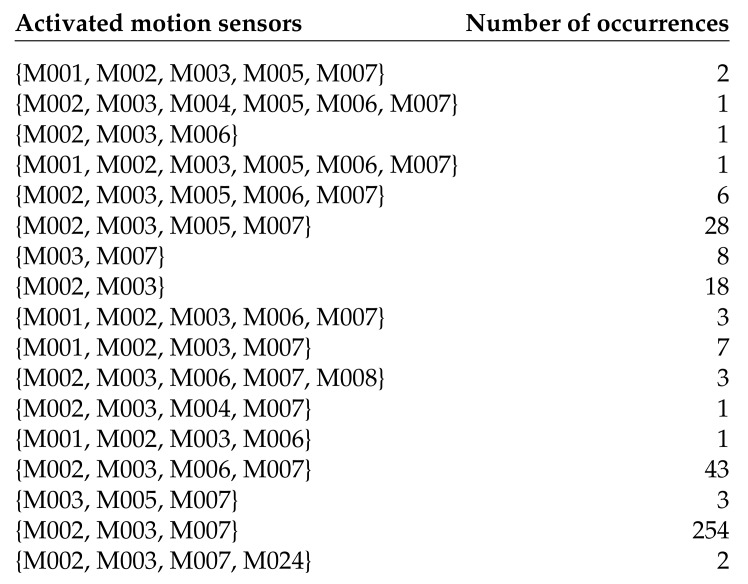
Sets of motion sensors activated by the sleeping activity. The analysis was performed on the Aruba data set.

We first preprocess the data set by making one pass through the data set during which we build a Python dictionary that shows for each type of activity the sets of motion sensors that have been activated. Since different occurrences of the same type of activity might trigger different motion sensors, we usually end up with more than one set of motion sensors. Figure 4 shows the dictionary entry for sleeping after preprocessing the Aruba data set.

After preprocessing the data set, we apply hierarchical clustering to the resulting dictionary as outlined in Section 3. For that we need to provide a cohesion value, which depends on the number of motion sensors installed, the overlap of the corresponding physical spaces, and other factors. Determining the best cohesion value is not straightforward, and therefore it is often necessary to run the clustering algorithm with different cohesion values.

## 7. Conclusions

This paper discusses how spatial information can be used explicitly to inform the activity recognition process in a smart home. The idea is to record the spatial markers that are triggered when an activity occurs and to compute lower and upper approximations of the set of markers for all recorded activities. The lower and upper approximations define rough sets, which are called activity spaces.

Since other approaches treat spatial information implicitly in the activity recognition process, there is no direct comparison between these and our approach. Nevertheless, we were able to obtain the following results:

- We developed a method that captures the spatial context in which activities occur in the form of XAI. The implementation of this method has linear time complexity.
- We showed that the method can boost the activity recognition process, depending on the layout of the smart home and the type of activity.
- We demonstrated how the method can help to distinguish between multiple residents living in the smart home.

When looking at the data sets that are commonly used to evaluate smart home algorithms, we can observe that they only record a very limited amount of different types of activities. With a small number of activities, it is likely that these happen in distinct places (e.g., sleeping in the bedroom, cooking in the kitchen, etc.), and as a result of that, have different activity spaces. Future work therefore requires more comprehensive data sets.
